# Supplementary material for: Splice-Junction-Based Mapping of Alternative Isoforms in the Human Proteome
Source: Cell Rep. Author manuscript; Available in PMC 2020 Jan 15. (PMC6961840; doi:10.1016/j.celrep.2019.11.026)
Supplement: 2 [file NIHMS1546469-supplement-2.zip › DF1/PXD010154/Liver/Liver_59_AKR1C2_LNDGHFMPVLGFGTYAPAELWSNS.pdf]

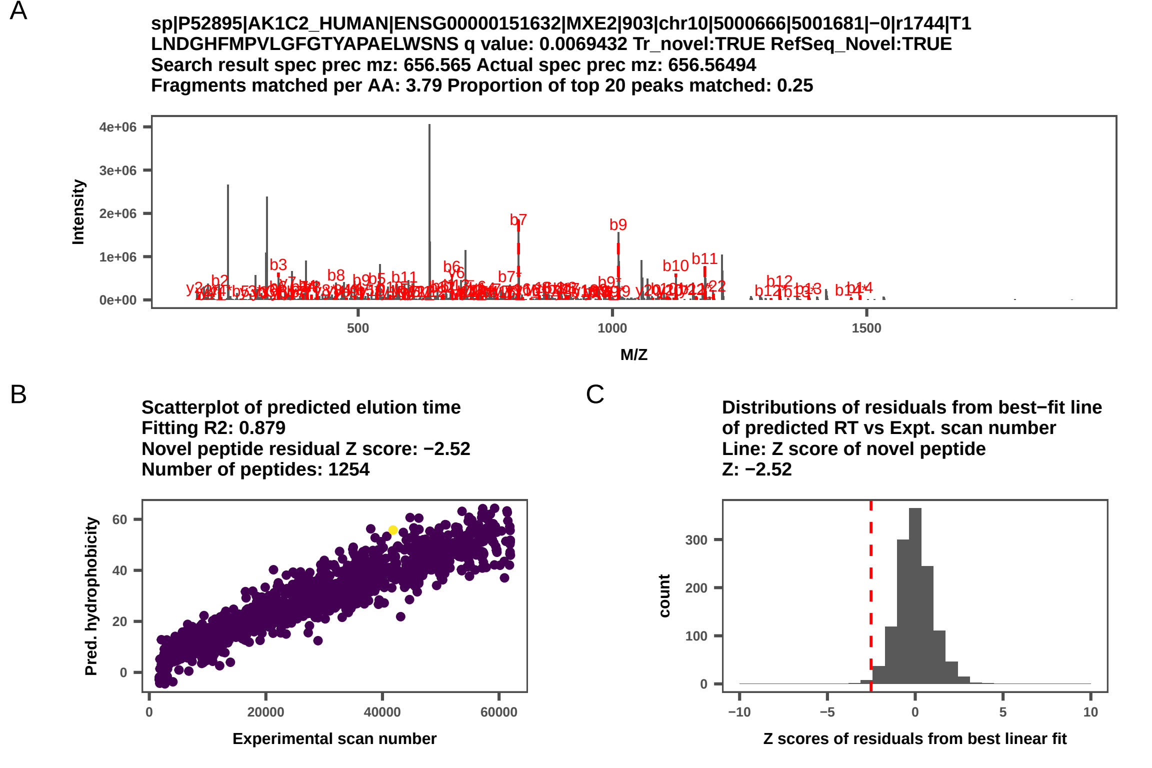
A
sp|P52895|AK1C2_HUMAN|ENSG00000151632|MXE2|903|chr10|5000666|5001681|−0|r1744|T1
LNDGHFMPVLGFGTYAPAELWSNS q value: 0.0069432 Tr_novel:TRUE RefSeq_Novel:TRUE
Search result spec prec mz: 656.565 Actual spec prec mz: 656.56494
Fragments matched per AA: 3.79 Proportion of top 20 peaks matched: 0.25
Intensity
4e+06
3e+06
2e+06
1e+06
0e+00
b7
b9
b3
b10
b11
b8
b6
y6
b7*
b12
b13
b14
500
1000
1500
M/Z
B
Scatterplot of predicted elution time
Fitting R2: 0.879
Novel peptide residual Z score: −2.52
Number of peptides: 1254
Pred. hydrophobicity
60
40
20
0
20000
40000
60000
Experimental scan number
C
Distributions of residuals from best−fit line
of predicted RT vs Expt. scan number
Line: Z score of novel peptide
Z: −2.52
count
300
200
100
0
−10
−5
0
5
10
Z scores of residuals from best linear fit
